# Adipose stem cells-derived small extracellular vesicles transport Thrombospondin 1 cargo to promote insulin resistance in gestational diabetes mellitus

**DOI:** 10.1186/s13098-024-01276-1

**Published:** 2024-05-19

**Authors:** Huaping Li, Hao Yang, Jingyan Liu, Hedi Yang, Xinyu Gao, Xiaoying Yang, Zhou Liu, Qiaohui Qian

**Affiliations:** 1 Department of Obstetrics and Gynecology, Shanghai University of Medicine & Health Sciences Affiliated Zhoupu Hospital, Shanghai, China; 2 Shanghai Key Laboratory of Molecular Imaging, Jiading District Central Hospital Affiliated Shanghai University of Medicine and Health Sciences, Shanghai, China; 3 Endocrinology Department, Shanghai University of Medicine & Health Sciences Affiliated Zhoupu Hospital, Shanghai, China

**Keywords:** Adipose-derived stem cells, Gestational diabetes mellitus, Insulin resistance, Small extracellular vesicles, Thrombospondin 1

## Abstract

**Background:**

Gestational diabetes mellitus (GDM) is a highly prevalent disease and poses a significant risk to the health of pregnant women. Abdominal adipose tissue (AT) contributes to insulin resistance (IR) associated with GDM. However, the underlying mechanisms remain unclear.

**Methods:**

In this study, we developed a mouse model of GDM by subjecting mice to a high-fat diet. We collected adipose-derived stem cells (ADSCs) from the abdominal and inguinal regions and examined their role in inducing IR in normal tissues through the secretion of small extracellular vesicles (sEVs). The sEVs derived from ADSCs isolated from GDM mice (ADSC/GDM) were found to inhibit cell viability and insulin sensitivity in AML12, a normal mouse liver cell line.

**Results:**

Through proteomic analysis, we identified high levels of the thrombospondin 1 (Thbs1) protein in the sEVs derived from ADSC/GDM. Subsequent overexpression of Thbs1 protein in AML12 cells demonstrated similar IR as observed with ADSC/GDM-derived sEVs. Mechanistically, the Thbs1 protein within the sEVs interacted with CD36 and transforming growth factor (Tgf) β receptors in AML12 cells, leading to the activation of Tgfβ/Smad2 signaling. Furthermore, the administration of LSKL, an antagonistic peptide targeting Thbs1, suppressed Thbs1 expression in ADSC/GDM-derived sEVs, thereby restoring insulin sensitivity in AML12 cells and GDM mice in vivo.

**Conclusions:**

These findings shed light on the intercellular transmission mechanism through which ADSCs influence hepatic insulin sensitivity and underscore the therapeutic potential of targeting the Thbs1 protein within sEVs.

**Supplementary Information:**

The online version contains supplementary material available at 10.1186/s13098-024-01276-1.

## Introduction

Gestational diabetes mellitus (GDM) refers to glucose intolerance that occurs or is first diagnosed during the second or third trimester of pregnancy [1]. It is considered an early stage of type 2 diabetes mellitus (T2DM). Recent changes in the population structure, including increased elderly fertility rates and obesity rates among women, have contributed to the rising prevalence of GDM has an upward trend in recent years [2]. The global prevalence of GDM varies substantially, ranging from 1% to > 30%, with Asians showing a particularly high prevalence (i.e., South-East Asia: 9.6–18.3%; Western Pacific (China): 4.5–20.3%) [3]. In China, due to the changing fertility strategy, the prevalence of high-risk pregnant women, such as those of advanced age and with pre-pregnancy overweight or obesity, has dramatically increased. A recent systematic review reported a pooled prevalence of GDM in China of 14.8% (95% confidence interval (CI) 12.8–16.7%) [4]. GDM poses significant risks of both the mother and the developing fetus. Up to 50% of GDM patients later develop T2DM [5], and affected mothers are at a greater risk of cardiovascular disease [6]. For infants, GDM can lead to neonatal hypoglycemia and insulin resistance (IR)-related obesity in young adulthood [7]. Additionally, approximately 35% to 50% of GDM progress to T2DM within 10 years after delivery [8].

The pathogenesis of GDM involves two main factors: high IR and decreased production of insulin by pancreatic β-cells [9]. Obesity and GDM are associated with elevated inflammatory markers, leading to IR. In obesity, rapid expansion of fat cells can result in lipid imbalance, chronic inflammation, and tissue fibrosis [10].Therefore, the role of inflammation in the development of GDM is considered crucial [11]. The liver, a target organ of IR-related diseases such as GDM and T2DM, exhibits dysfunction in the early pregnancy, which can increase the risk of GDM in the pregnancy period [12, 13].

Adipose-derived stem cells (ADSCs) are a type of mesenchymal stem cells obtained by separating adipocytes. They possess the ability to differentiate into other adipocytes and secrete various paracrine cytokines, growth factors, microRNAs, and important components such as small extracellular vesicles (sEVs) that support normal cellular functions. The potential therapeutic applications of sEVs derived from ADSCs have been confirmed for "stem cell-free therapy" in neurodegenerative and metabolic diseases [14]. However, factors such as a long-term hyperglycemic environment and reactive oxygen species within the recipient can alter the biological function of ADSCs [15]. Currently, there are no reports of abnormal expression of sEVs derived from ADSCs under pathological conditions. Nevertheless, evidence suggests that sEVs isolated from plasma of GDM females can induce abnormal glucose tolerance and impair skeletal muscle sensitivity to insulin in mice [16].

The endoplasmic reticulum (ER), an organelle responsible for storing calcium ions, plays a crucial role in synthesizing and processing membrane proteins and lipid biosynthesis. Maintaining ER's internal environment stability is vital for cellular survival, proliferation, and growth [17]. Thrombospondin 1 (Thbs1), a protein secreted by small sEVs from ADSCs, possesses multiple functional domains and is predominantly expressed in the visceral adipose tissue (AT) of IR or obese individuals [18]. GDM can activate the ER stress signal, disrupt ER homeostasis, promote the accumulation of unfolded proteins within the ER lumen, and induce the unfolded protein response or ER stress (ERS). ERS plays a central role in triggering IR and T2DM [19].

Thbs1 has been identified as one of the markers in the early stages of diabetes and a key mediator in its development. The peptide antagonist, LSKL, can inhibit Thbs1 activity, thereby reducing diabetes-related complications resulting from Thbs1 upregulation [20, 21]. The expression of Thbs1 is positively correlated with obesity and IR. Knocking out Thbs1 in AT can inhibit tissue inflammation caused by obesity and improve tissue insulin sensitivity [22]. However, little is known about the abnormal expression of ADSC-derived exosomes under GDM conditions and how they affect the insulin sensitivity of GDM target organs, particularly the liver histiocyte, promoting the development of IR in GDM. Therefore, our study aimed to investigate the intercellular transmission mechanism of ADSCs that influence insulin sensitivity.

## Materials and methods

### Establishment and identification of mice with GDM

Five-week-old female C57BL/6 J mice were purchased from Shanghai Bikaikeyi Biotechnology Co., LTD. (License No. SCXK2018-0006) and housed in an SPF animal laboratory. Prior to the experiment, tail venous blood samples were collected from the mice to determine fasting blood glucose levels, with a normal range of 4–7 mmol/L. Sixteen mice were randomly divided into two groups: one group received a high-fat diet containing 60% fat (XTHF60, Xietong pharmaceutical bio, Nanjing, China; 8 mice), while the other group received a normal diet (8 mice). After 8 weeks, female mice were placed in the same cage with male C57BL/6 J mice at a ratio of 2:1. Once pregnancy was confirmed, the mice continued to be fed their respective diets for 12 days. Blood glucose levels were recorded every 3 days during this period, and GDM was considered successfully induced if random blood glucose levels exceeded 11.1 mmol/L. On day 12, plasma was collected, and the concentrations of insulin, leptin, adiponectin, and hypersensitive C-reactive protein (hs-CRP) were determined using an enzyme-linked immunosorbent assay (ELISA) kit (Mlbio, Shanghai, China) following the manufacturer's instructions. All animal experiments were approved by the Animal Ethics Committee of Shanghai University of Medicine and Health Sciences.

### ADSCs isolation and cell culture

Primary adipose stem cells (ADSCs) were isolated from GDM mice and normal gestational mice, respectively. Under aseptic conditions, the abdominal cavity of the mice was opened, and AT from the abdominal and inguinal regions was obtained. The AT was then rinsed, minced, and collected in a pre-cooled Hank’s Balanced Salt Solution (Sangon BIOTECH, Shanghai, China). Next, 2 mg/ml collagenase I (Yeason, Shanghai, China) and 3 mM CaCl_2_ were added in double volume to the tissue, which was then digested at 37 ℃ for 4 h. Digestion was stopped by adding an equal volume of DMEM/F12 medium (Gibco, GIBCO, NY, USA) containing 10% FBS (Merck KGaA, Darmstadt, Germany), followed by centrifugation at 1200 g for 10 min. The cell precipitates were resuspended, washed with PBS, and cultured in DMEM/F12 medium containing 10% FBS. Mouse normal liver cells, AML12, were purchased from Shanghai Fuheng Biotechnology and cultured in DMEM/F12 medium containing 10% FBS, 1% ITS media supplement (R&D Systems, MN, USA), and 40 ng/ml dexamethasone (Merck KGaA).

### Plasmid and reagent

The encoding region of mouse Thbs1 mRNA (NCBI number: NM011580) was cloned onto the pcDNA3.1–3 × Flag-eGFP-C2 vector. The control vector and Flag-eGFP-Thbs1 fusion plasmid were transfected into AML12 cells using liposomal transfection reagent (Yeason). Thbs1 pharmacological inhibitory peptide LSKL and transforming growth factor (Tgf) β inhibitor ITD-1 were purchased from Selleck Chemicals (Shanghai, China).

### Extraction, purification and identification of sEV

When the confluency of ADSCs reached 80–90%, the medium was changed to DMEM/F12 medium containing 10% FBS without sEVs and continued to culture for 72 h. The supernatant was then centrifuged at a low speed, and large vesicles were removed by filtration using a 0.22 μm pore size filter. The culture medium was subjected to ultracentrifugation at 120,000 × g for 90 min (OptimaTM XPN-100, Beckman Coulter, USA). The resulting sEV precipitates were resuspended in pre-cooled PBS, followed by another round of centrifugation. Subsequently, the sEV precipitates were resuspended in an appropriate volume of phosphate buffered saline (PBS) and characterized using nanoparticle tracking analysis (NTA) with ZetaView PMX 110 (Particle Metrix, Meerbusch, Germany). The sEV solution was placed on copper grids (Zhongjingkeyi Technology, Beijing, China), stained with 50 μL of uranium acetate, and visualized using transmission electron microscopy (FEI Tecnai G2 Spirit TEM, USA) for sEV visualization.

### Western blotting

AML12 cells were lysed using radioimmunoprecipitation assay (RIPA) buffer (KeygenBio, Nanjing, China) containing a protease inhibitor cocktail (P8340, Merck KGaA). Total proteins in AML12 cell lysates or sEV solutions were quantified using a bicinchoninic acid (BCA) protein assay kit (Yeason). Ten micrograms of protein were resolved on SDS polyacrylamide gel before being transferred to a PVDF membrane (Millipore, USA). The membrane was then blocked with 5% bovine serum albumin (BSA, Sangon BIOTECH) at room temperature for 1 h, incubated with primary antibodies overnight at 4℃, and subsequently incubated with secondary antibody for 1 h. Images were captured using a Bioanalytical imaging system (Tanon 5200 Multi system, Shanghai, China). The primary antibodies used were as follows: anti-Cd63 (25682-1-AP, Proteintech, Wuhan, China), anti-Tsg101 (72312, Cell Signaling Technology, MA, USA), anti-Thbs1 (37879, Cell Signaling Technology), anti-Cd44 (15675-1-AP, Proteintech), anti-Phospho-Jnk (AP0631, ABclonal, Wuhan, China), anti-Jnk (A4867, ABclonal), anti-Atf4 (A0201, ABclonal), anti-Atf6 (A0202, ABclonal), anti-Ire1 (A17940, ABclonal), anti-Grp78 (A4908, ABclonal), anti-Chop (A0221, ABclonal), anti-Smad2 (A7699, ABclonal), anti-Phospho-Smad2 (AP0269, ABclonal), anti-Tgfβ2 (19999-1-AP, Proteintech), and anti-CD36 (A17339, ABclonal).

### Immunofluorescence

A total of 2 × 10^4^ ADSC or ADSC/GDM cells were cultured in 24-well plates with preset glass plates. After cell adhesion, they were fixed for 30 min in 4% paraformaldehyde. Subsequently, the cells were sealed in a PBS solution containing 0.5% Triton X-100 and 10% fetal bovine serum (FBS) for 2 h. The glass slides were then incubated with primary Cd44 antibody and Alexa Fluor 594-labeled secondary antibody (SA00006-4, Proteintech). Following the cleaning process with PBS, the slides were stained with 4′,6-diamidino-2-phenylindole (DAPI)solution for 5 min. Finally, the slides were embedded in fluoromount-G (SouthernBiotech, USA) and photographed using a fluorescence microscope (DS-Ri2, Nikon, Japan).

### Cell viability assay

A total of 10^4^ normal mouse liver cells (AML12) were seeded in 96-well plates. Once adhered, sEVs with final concentrations of 0, 10^8^, 5 × 10^8^, and 10^9^ particle numbers/mL were added to serum-free DMEM/F12 medium and treated for 48 or 72 h. Each well was supplemented with 100 μL of culture medium and 10 μL of CCK8 reagent (Laisi biotech, Shanghai, China). The cell viability was measured by microplate reader, determining the OD450nm value.

### Cell apoptosis assay

AML12 cells at a density of 8 × 10^5^ were placed in a 6-well plate and co-cultured with sEVs at a concentration of 10^8^ particle numbers/mL for 48 or 72 h. Cell apoptosis was detected using propidium iodide (PI) staining and the Annexin V apoptosis detection kit (BD Biosciences, NZ, USA) via flow cytometry (Novocyte, Agilent Technologies, CA, USA). The proportion of apoptotic cells was analyzed using NovoExpress software (version 1.5.0, Agilent Technologies).

### Insulin sensitivity assay

A total of 6 × 10^5^ AML12 cells were seeded in a 12-well plate. After cell adhesion, the AML12 cells were incubated in serum-free medium with sEVs at a concentration of 10^8^ particles/mL for 48 or 72 h. In the control wells, the same volume of serum-free medium was added. Following co-culture, the AML12 cells were treated with or without 1 μg/mL insulin for 1 h. Glucose content was determined by collecting the culture medium and using the glucose detection kit (GAGO20, Merck KGaA). Glucose uptake was measured by subtracting the glucose level in the cultured well from that in the cell-free well, which reflected the insulin sensitivity.

### Real-time polymerase chain reaction (PCR)

Total RNA was isolated from AML12 cells co-cultured with sEVs using the RNeasy Mini kit (Qiagen, Hilden, Germany). Synthesis of complementary DNA (cDNA) was performed using HiScript II Q RT SuperMix (Vazyme, Nanjing, China). Real-time PCR was conducted on QuantStudio 7 (Thermo Fisher Scientific) using SYBR Premix Ex Taq (Takara, Otsu, Japan). Gene expression was quantified using delta Ct. The primer sequences for all mRNA are provided in “Additional file 3: Table S1”.

### Proteomics of sEV

Differences in expression of the sEV proteome secreted by normal ADSCs and GDM mice-derived ADSCs were identified using label-free protein quantification. The proteins in the sEVs were extracted and analyzed by nano-high-performance liquid chromatography-tandem mass spectrometry (HPLC–MS/MS, Thermo Q Exactive). The data acquisition mode was data-dependent acquisition (DDA). The series of mass spectra were analyzed using PEAK Studio version X (Bioinformatics Solutions Inc., Waterloo, Canada), and protein databases were searched using PEAK DB. These detection procedures were performed by Guangzhou Gene denovo Biotechnology Co., Ltd. (Guangzhou, China).

### Co-immunoprecipitation

AML12 cells treated with sEVs or inhibitors were lysed with RIPA buffer. AML12 cell extracts were immunoprecipitated with anti-Tgfβ2 or anti-CD36 antibodies for 24 h, then co-incubated with protein A/G magnetic beads (Bimake, Shanghai, China) for 3 h. The magnetic beads carrying interacting proteins were heated at 100 ℃ for 5 min, and the expression levels of interacting proteins were analyzed by western blotting.

### Histological analysis

Liver tissue, abdominal AT, and inguinal AT from normal mice, GDM mice, and GDM mice treated with LSKL were fixed with 4% paraformaldehyde and embedded in paraffin. AT and liver tissue morphology were observed through hematoxylin–eosin (HE) staining. For immunohistochemistry (IHC), tissue sections were sequentially dewaxed, rehydrated, and treated with 3% H_2_O_2_. Sections were blocked in 5% BSA for 30 min, and then incubated overnight with anti-Tsg101, Thbs1, p-Jnk, Atf6, Ire1, and p-Smad2 antibodies at 4 ℃. After incubation with an enzyme-labeled secondary antibody at room temperature, the positive signal was observed using diaminobenzene (DAB) chromogenic agent and imaged under an optical microscope.

### Statistical analysis

Data analysis was performed using GraphPad Prism 7.0 (GraphPad Software, USA) and SPSS 20.0 software (SPSS Inc., USA). Results are expressed as mean ± standard deviation (SD). Student’s t-test was used to analyze statistical differences between two groups of samples. One-way analysis of variance (ANOVA) and Bonferroni post hoc tests were used to analyze statistically significant differences among three or more groups. A *P*-value of < 0.05 was considered statistically significant.

## Results

### Isolation of adipose stem cells and sEV from mice with GDM

**Fig. 1:**
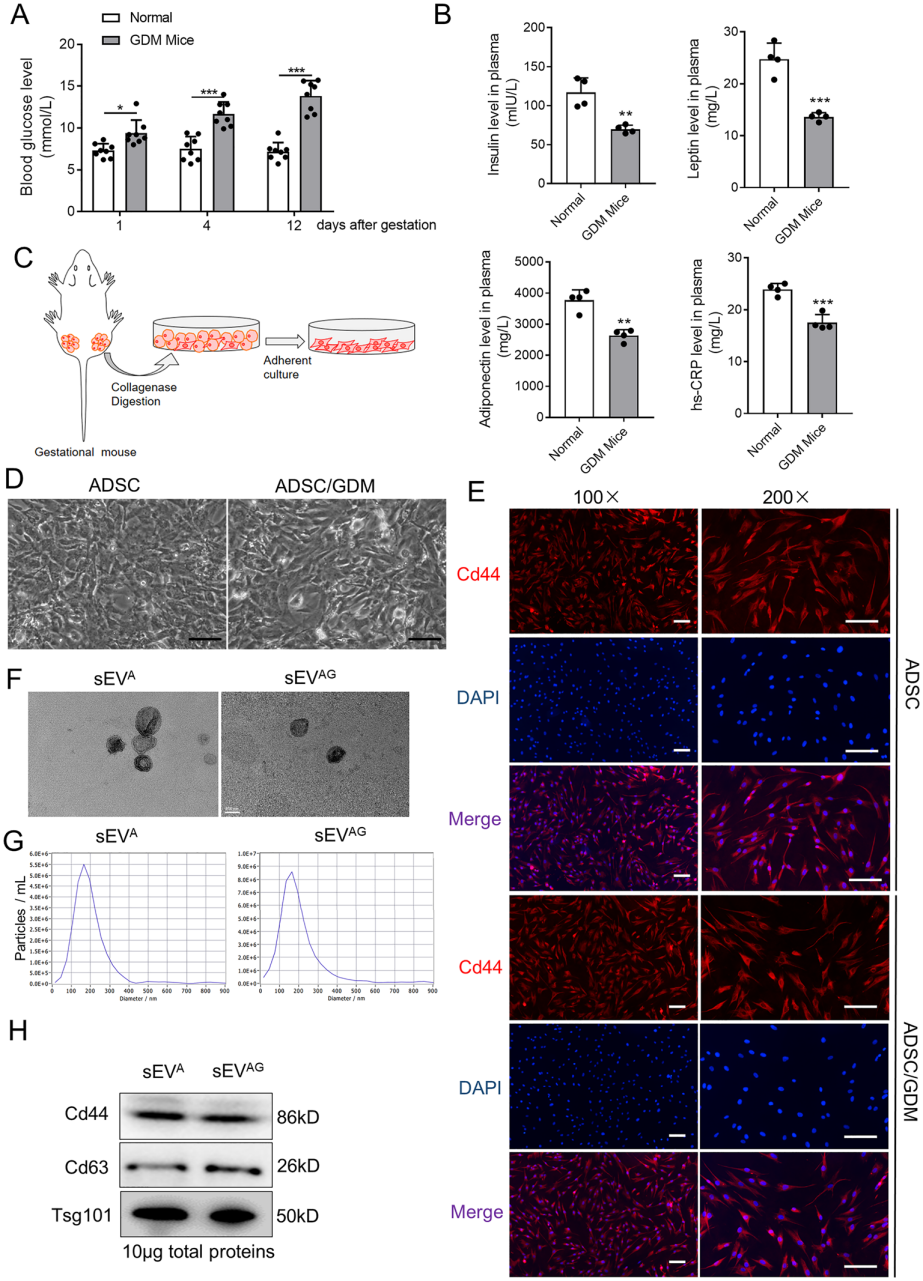
Isolation, culture and purification of GDM mice-derived ADSCs and their sEVs. **A** Plasma glucose values were quantified in eight normal gestation and GDM mice. **B** The expression of insulin, leptin, adiponectin and hs-CRP in the plasma of mice was detected by ELISA assays. **C** ADSCs was obtained by collagenase digestion of abdominal and inguinal adipose tissue from gestational mice and further in vitro culture. **D** Microscopic images of ADSCs isolated and cultured from normal gestational and GDM mice. Scale bar, 25 μm. **E** The expression of Cd44 marker (red) was identified by immunofluorescence. DAPI staining (blue) indicated the nucleus. Scale bar, 25 μm. **F** The sEV^A^ and sEV^AG^ were identified by transmission electron microscope. Scale bar, 100 nm. **G** The sEV^A^ and sEV^AG^ solution was diluted 500 times and particle size distribution and concentration were determined by nanoparticle tracking analysis. **H** The sEV markers and stem cell markers were detected by western blotting in sEV^A^ and sEV.^AG^ samples. Data were presented with mean ± standard deviation (SD), *P < 0.05, **P < 0.01, ***P < 0.001

High fat fed C57BL/6 J gestational mice were used as GDM models, monitoring their blood glucose levels during gestation. We observed a significant increase in blood glucose levels in GDM mice compared to normal gestational mice at day 1, 4, and 12 after gestation “Fig. 1A”. The plasma of mice at day 12 of gestation was then determined by enzyme linked immunosorbent assay (ELISA). Compared with normal gestational mice, a significant decrease in insulin, leptin, adiponectin, and hypersensitive C-reactive protein (hs-CRP) levels in the plasma of GDM mice was observed “Fig. 1B”. This finding suggests that GDM mice exhibit reduced insulin sensitivity in comparison to normal gestational mice. In order to investigate the role of AT in the functionality of other tissues and insulin sensitivity in GDM mice, we isolated primary adipose stem cells from the abdominal and inguinal AT of GDM mice (ADSC/GDMs), as well as from normal gestational mice (ADSCs) “Fig. 1C, D”. Immunofluorescence analysis confirmed positive expression of the stem cell marker Cd44 in both isolated primary ADSCs and ADSC/GDMs, alongside Cd29, a mesenchymal stem cell markers (Fig. 1E and Additional file 1: Figure S1). Conversely, the monocyte population marker Cd14 showed negative expression in both ADSCs and ADSC/GDMs (Additional file 2: Figure S2). Subsequently, we cultured and subcultured these primary ADSCs and ADSC/GDMs on a large scale and isolated and purified sEVs from the cell culture medium via a standard ultracentrifugal procedure. Transmission electron microscopy (TEM) and nanoparticle tracking analysis (NTA) were employed to determine the morphology, size, and concentration of sEVs derived from purified ADSCs (sEV^A^) and ADSC/GDMs (sEV^AG^) “Fig. 1F, G”. Western blotting analysis confirmed the expression of stem cell marker protein Cd44, as well as extracellular vesicle marker proteins Cd63 and Tsg101, in sEV^A^ and sEV^AG^ “Fig. 1H”. Consequently, we successfully isolated ADSCs from both GDM mice and normal gestational mice and purified their respective sEVs.

### ADSC/GDM-derived sEV inhibited IR in normal liver cells

**Fig. 2:**
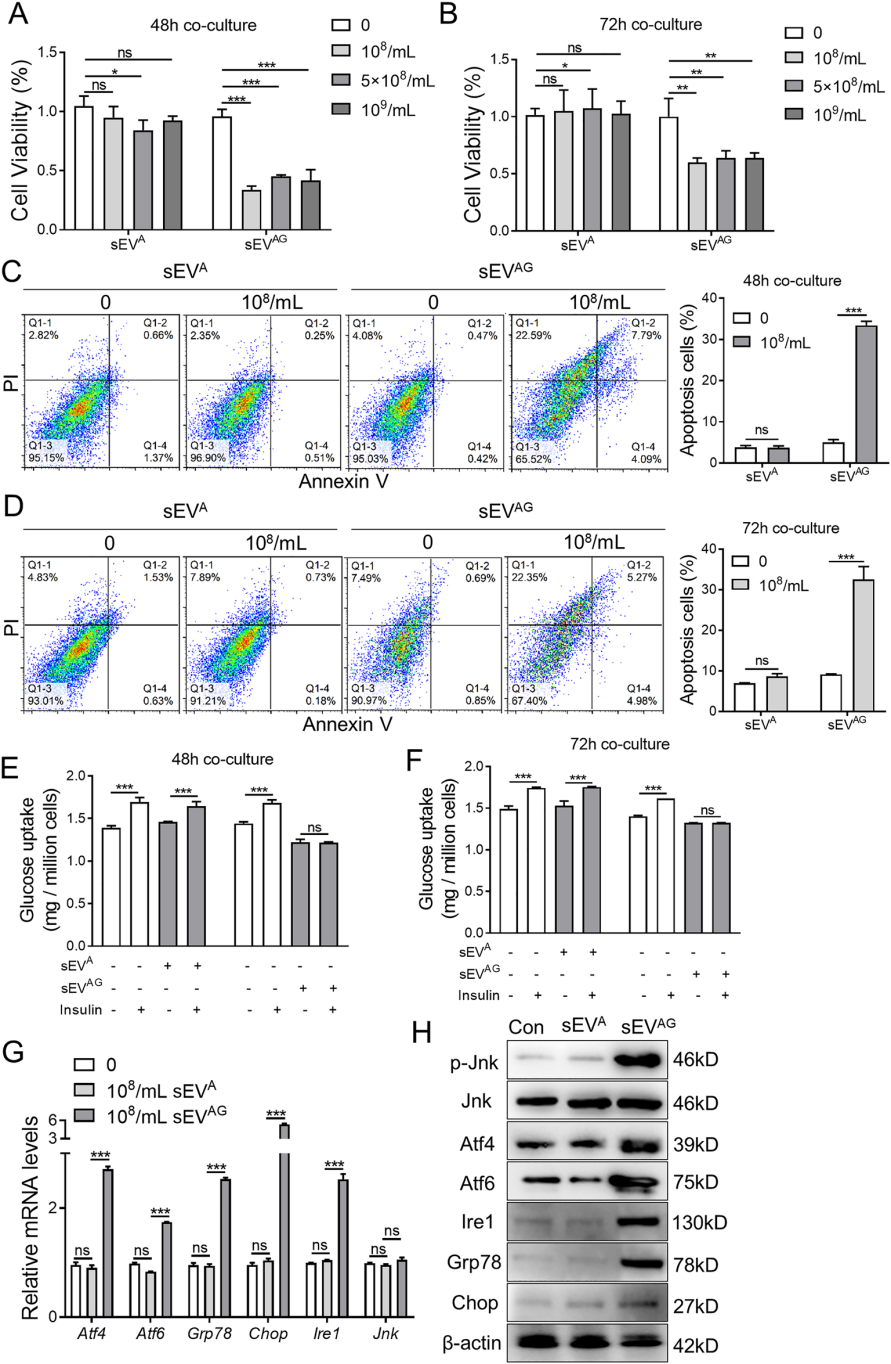
ADSC/GDM-derived inhibits hepatocyte proliferation and insulin sensitivity in normal mice. **A**, **B** The sEV^A^ and sEV^AG^ with different concentrations were co-cultured with AML12 cells for 48 or 72 h. Cell viability was obtained by CCK8 assays. **C**, **D** The sEV^A^ and sEV^AG^ with 10^8^ particles / mL were co-cultured with AML12 cells for 48 or 72 h. Cell apoptosis was detected by flow cytometry. **E**, **F** The sEV^A^ and sEV^AG^ with 10^8^ particle number / mL were co-cultured with AML12 cells for 48 or 72 h, then cells were treated with 1 μg/ml insulin for 1 h. Glucose uptake was measured to reflect insulin sensitivity of AML12 cells in each treatment. **G** The sEV^A^ and sEV^AG^ with 10.^8^ particle number /mL were co-cultured with AML12 cells for 48 h, and mRNA expression of endoplasmic reticulum (ER)-related genes was detected by RT-PCR. **H** The expression of ER-related proteins was detected by western blotting. Data were presented with mean ± standard deviation (SD); ns, no significance; *P < 0.05, **P < 0.01, ***P < 0.001

We hypothesized that ADSC/GDMs in AT affect insulin sensitivity in other tissues through the secretion of sEVs. The sEV^A^ and sEV^AG^ were co-cultured with normal mouse liver cells (AML12) to assess changes in cell viability, apoptosis, and insulin sensitivity. At different concentrations and time points, sEV^A^ derived from normal gestational mice had little effects on the viability of AML12 cells “Fig. 2A and B”. Conversely, sEV^AG^ obtained from GDM mice significantly inhibited AML12 cell viability “Fig. 2A, B”. We selected a concentration of 10^8^ particles/mL for sEV^A^ and sEV^AG^ co-cultured with AML12 cells for 48 and 72 h to evaluate cell apoptosis. The results demonstrated that sEV^AG^ significantly promoted apoptosis of AML12 cells, while sEV^A^ did not induce such effects “Fig. 2C, D”. Subsequently, we stimulated AML12 cells with insulin after co-culturing them with sEV^A^ and sEV^AG^, and evaluated glucose metabolism in these cells. The findings revealed a significant increase in glucose uptake in control AML12 cells and sEV^A^ co-cultured AML12 cells after insulin stimulation at 48 and 72 h of co-culture. Conversely, insulin failed to regulate glucose uptake in sEV^AG^ co-cultured AML12 cells “Fig. 2E, F”. These results indicate that sEV^AG^ inhibits insulin sensitivity in AML12 cells. Previous comprehensive studies have suggested a close association between gestational diabetes-induced IR and ERS [17, 23, 24]. To further investigate the effects of sEV^A^ and sEV^AG^ on ER stress-related regulatory pathways, we conducted RT-PCR “Fig. 2G” and western blotting “Fig. 2H” analyses. The data revealed that sEV^AG^ significantly increased the expression of ER stress-related genes and proteins, including phosphorylated Jnk, Atf4, Atf6, Ire1, Grp78, and Chop, in AML12 cells compared to normal AML12 cells or those co-cultured with sEV^A^. Consequently, sEV^AG^ secreted by ADSCs derived from GDM mice impairs viability and insulin sensitivity in normal hepatocytes while promoting ERS.

### Proteomics revealed high Thbs1 expression in ADSC/GDM-derived sEV

**Fig. 3:**
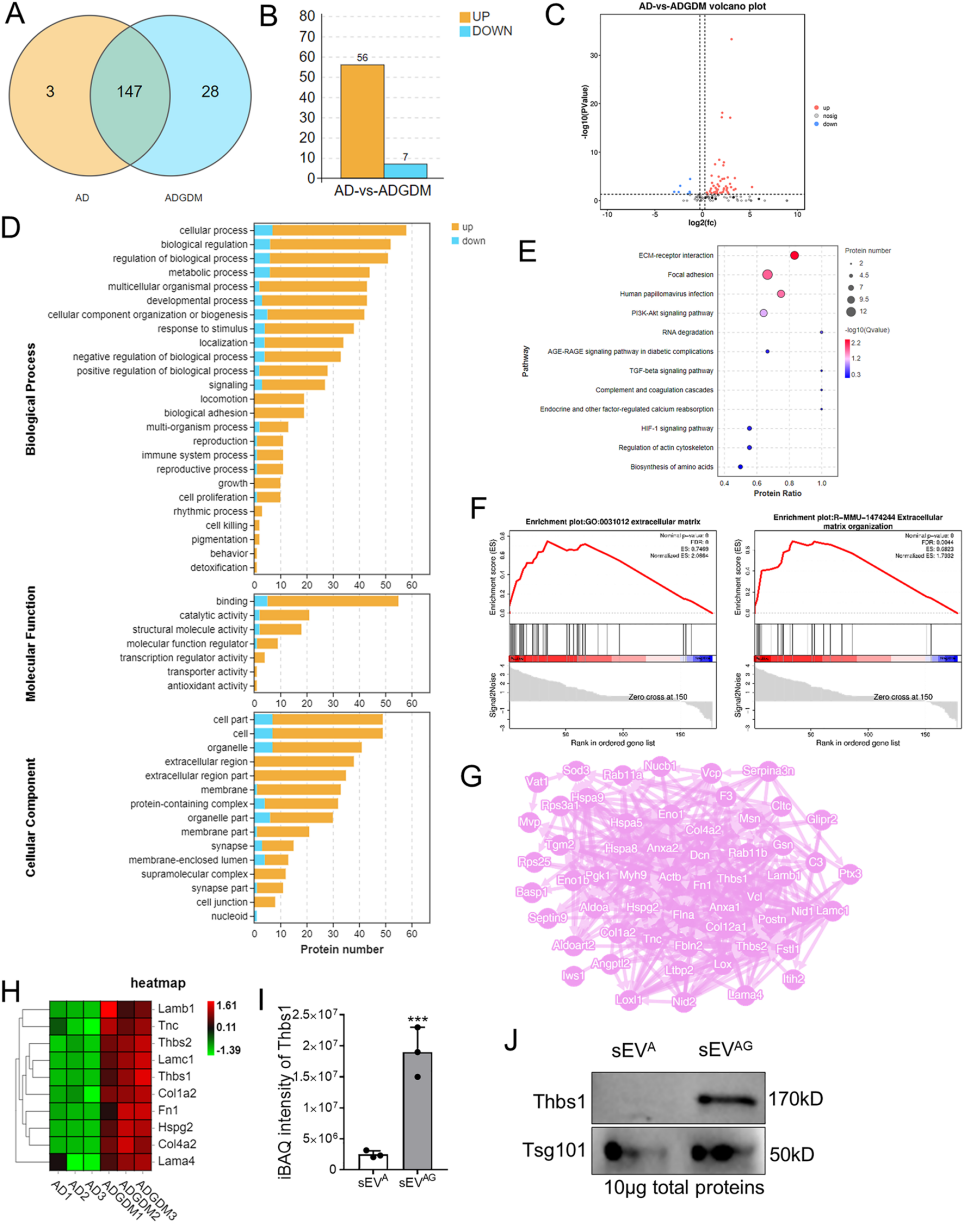
Proteomic identification of high Thbs1 expression in ADSC/GDM-derived sEV. **A** Venn diagram of the number of proteins detected by unlabeled protein profiles for and sEV^A^ (AD in graph) and sEV^AG^ (ADGDM in graph). **B** The number of proteins with high and low expression in sEV^AG^. **C** Volcanic map of differentially expressed protein in sEV^AG^. **D** GO signaling pathway analysis of sEV^A^ and sEV^AG^ proteins. **E** KEGG signal analysis of sEVA and sEV^AG^ proteins. **F** GSEA analysis revealed enrichment of the ECm-receptor interaction pathway. **G** Network analysis of interactions between differential ECM regulatory proteins and other differential proteins. H. Heat maps of 10 proteins significantly overexpressed in sEV^AG^. **I**, **J** Mass spectrometry quantification and western blotting of Thbs1 in sEV^A^ and sEV.^AG^. ***P < 0.001

To unravel the mechanism underlying sEV^AG^-mediated IR, we conducted a comparative analysis of the proteomes of sEV^A^ and sEV^AG^ using protein mass spectrometry. In total, we identified 462 peptides and 178 proteins in six sEV samples (three replicates per group) from the sEV^A^ and sEV^AG^ groups. Out of these, 147 proteins were expressed in both groups of sEV samples “Fig. 3A”. We further analyzed the 147 proteins expressed in both groups using R language and Student's t-test for quantification, resulting in the identification of differentially expressed proteins for each comparison group. The screening conditions included a *P*-value less than 0.05 obtained through the t-test, a fold-change greater than 1.2, and the presence of at least one unique peptide segment. Consequently, we identified 63 differential sEV proteins. Comparing the sEV^A^ and sEVAG groups, 56 proteins were significantly overexpressed in the sEV^AG^ group, while 7 proteins were significantly under-expressed “Fig. 3B, C, Additional file 4: Table S2″. Gene Ontology (GO) analysis revealed that these differentially expressed proteins were involved in both intracellular and extracellular life activities “Fig. 3D”. KEGG bubble map analysis demonstrated that proteins associated with extracellular matrix (ECM)-receptor interactions exhibited the most significant differences in the sEV^AG^ group (*P* = 0.00008), “Fig. 3E”. Gene set enrichment analysis (GSEA) showed an enrichment of ECM-mediated intercellular communication proteins in sEV^AG^ “Fig. 3F”. Additionally, the Search Tool for the Retrieval of Interacting Genes (STRING)protein interaction network analysis displayed interactions between hub proteins responsible for ECM and cell communication”Fig. 3G”. Among the proteins involved in regulating ECM-receptor interactions, 10 proteins exhibited the most significant differences in sEV^AG^ “Fig. 3H”. Notably, Thbs1 displayed the most significant difference between the two groups “Fig. 3I”, (*P* = 2.67E-16). We further confirmed the high expression of Thbs1 in sEVAG using western blotting “Fig. 3J”. Therefore, proteomic analysis unveiled the contents of sEVAG and indicated that the high expression of Thbs1 may contribute to IR.

### Thbs1 damaged insulin sensitivity of normal liver cells

**Fig. 4:**
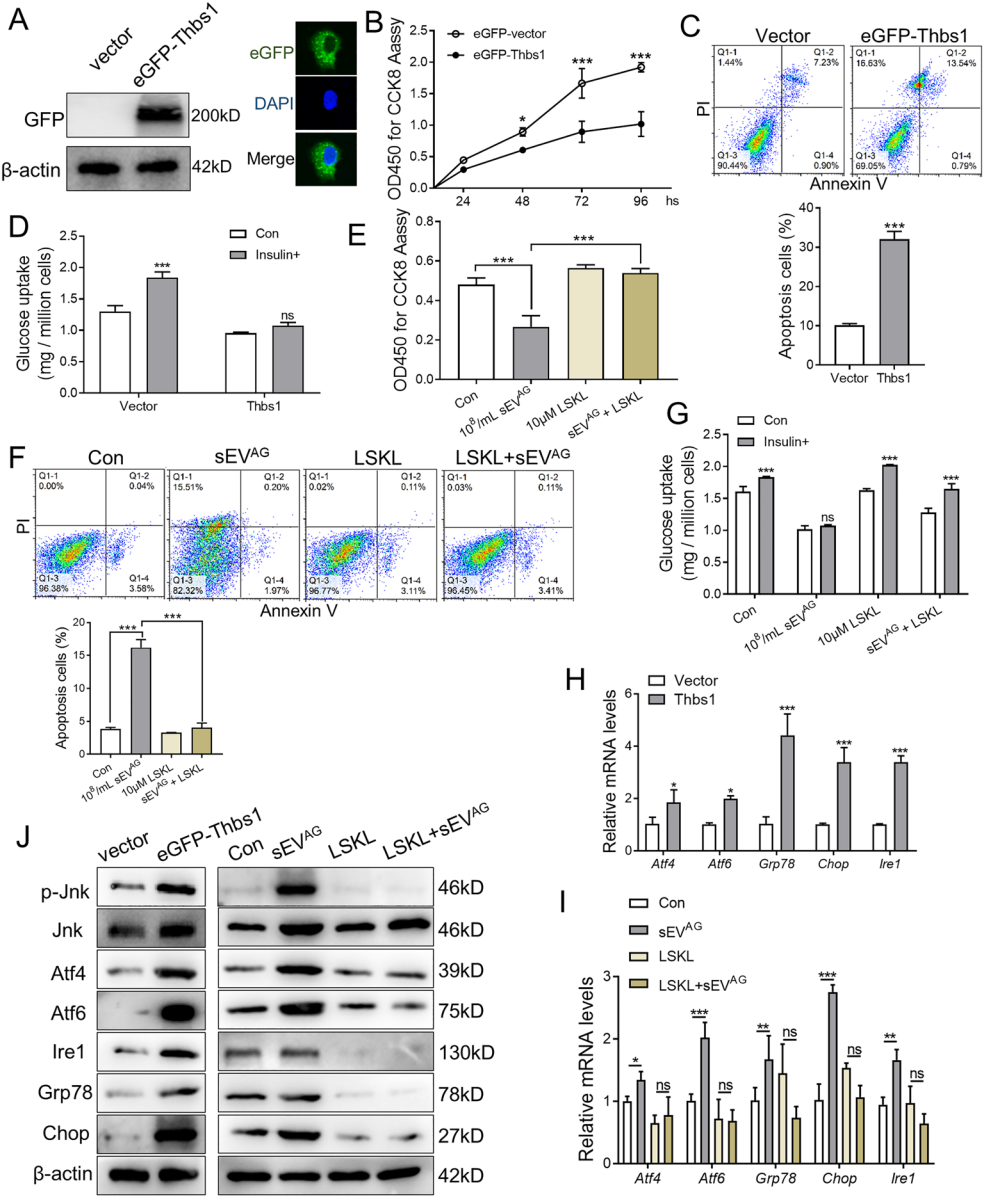
Overexpression of Thbs1 inhibits insulin sensitivity in AML12 cells. **A** Flag-eGFP-Thbs1 plasmid and control vector were transfected into AML12 cells. The expression of eGFP-thbs1 fusion protein in AML12 was identified by western blotting and fluorescence microscopy. **B** Plasmid transfected AML12 cells were placed in 96-well plates, and cell viability was detected by CCK8 assay. **C**. Apoptosis assays of AML12 cells after plasmid transfection were performed. **D** Plasmid transfected AML12 cells were treated with 1 μg/ml insulin for 1 h, and the culture medium was collected for glucose uptake measurement. **E**–**G** AML12 cells were treated with 10^8^/mL sEV^AG^, 10 μM LSKL and 10^8^/mL sEV^AG^ + 10 μM LSKL for 48 h. Cell viability, apoptosis, and insulin-induced glucose uptake were then measured. **H** The expression of endoplasmic reticulum stress (ERS)-related mRNA in AML12 cells after plasmid transfection was detected by RT-PCR. **I** The expression of ERS-related mRNA in 10^8^/mL sEV^AG^ and/or 10 μM LSKL treated AML12 cells was detected by RT-PCR. **J** The expression of ERS-related proteins in AML12 cells was detected by western blotting. Statistical data were presented as mean ± standard deviation (SD); ns, no significance; *P < 0.05, **P < 0.01, ***P < 0.001

To investigate the impact of Thbs1 on the viability and insulin sensitivity of mouse liver cells, we transfected AML12 cells with Flag-eGFP-Thbs1 plasmid and a control vector. The expression of Thbs1 was confirmed in the transfected AML12 cells by detecting the GFP fusion protein “Fig. 4A”. Overexpression of Thbs1 significantly compromised the viability of AML12 cells “Fig. 4B” and increased cell apoptosis “Fig. 4C”. In AML12 cells with elevated Thbs1 expression, insulin exhibited limited effectiveness in facilitating glucose uptake, indicating insulin insensitivity “Fig. 4D”. To counteract the IR induced by sEV^AG^ and Thbs1, AML12 cells were treated with LSKL, an inhibitory polypeptide specific to Thbs1. The results demonstrated that LSKL significantly improved cell viability “Fig. 4E” and reduced the percentage of apoptotic cells”Fig. 4F” in sEV^AG^-pretreated AML12 cells. Moreover, AML12 cells treated with sEV^AG^ and LSKL exhibited restored insulin-induced glucose uptake “Fig. 4G”. In terms of ER stress signaling related to the regulation of insulin sensitivity, Thbs1 overexpression in AML12 cells resulted in high mRNA levels of *Atf4, Atf6, Ire1, Grp78,* and *Chop* “Fig. 4H and I”, as well as increased protein expressions of phosphorylated Jnk, Atf4, Atf6, Ire1, Grp78, and Chop “Fig. 4J”. However, after LSKL treatment, the expression of these markers was suppressed. Consequently, the elevated expression of Thbs1 in sEV^AG^ inhibited the viability and insulin sensitivity of mouse liver cells.

### Thbs1 from ADSC/GDM sEV binded to CD36 receptor and activated Tgfβ1 signaling

**Fig. 5:**
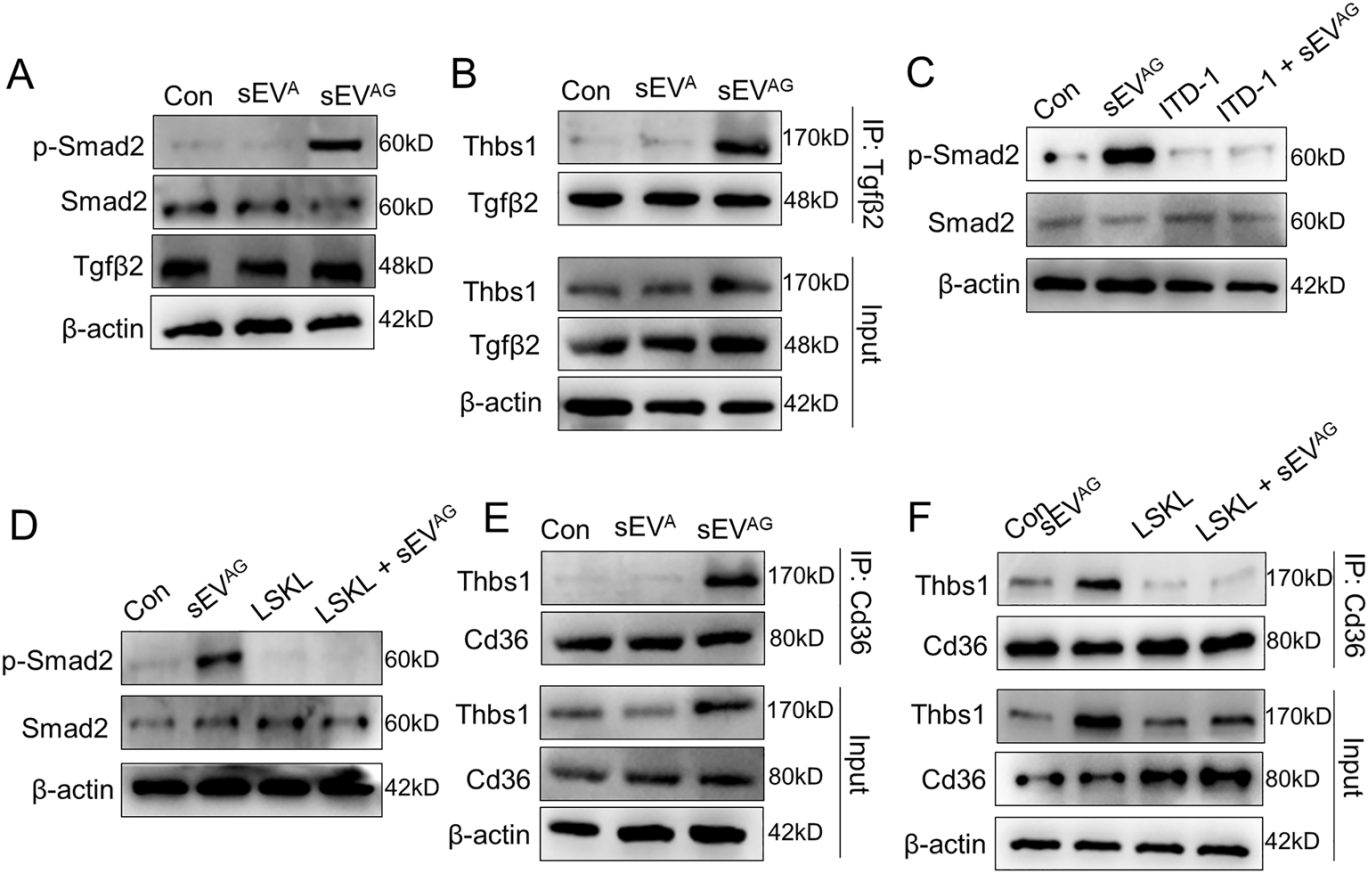
ADSC/GDM-derived sEV carrying Thbs1 induces Tgfβ and CD36 activation. **A** sEV^AG^ was co-cultured with AML12 for 48 h, and the expression of Smad2, phosphorylated Smad2 and Tgfβ was detected by western blotting. **B** sEV^AG^ was co-cultured with AML12 for 48 h, and the cell lysate was subsequently subjected to co-immunoprecipitation (co-IP) with anti-Tgfβ antibodies. Thbs1 levels in co-IP products and input samples were detected. **C, D** AML12 cells were treated with 108 /mL sEV^AG^, 2 μM ITD-1, 10 μM LSKL or a combination for 48 h. The expression of Smad2 and phosphorylated Smad2 was detected by western blotting. **E** Cell lysate was co-immunoprecipitated with anti-CD36 antibody. Thbs1 levels in co-IP products and input samples were detected. **F** AML12 cells were treated with 10^8^/mL sEV^AG^, 10 μM LSKL or a combination for 48 h, followed by co-IP with anti-CD36 antibody

Thbs1, a membrane protein abundant in AT, triggers the Tgfβ/Smads signaling pathway [25]. LSKL functions as a competitive antagonist that impedes the activation of Thbs1/Tgfβ by inhibiting the interaction between the KRFK sequence of Thbs1 and Tgfβ [26]. We hypothesized that Thbs1 present in sEV^AG^ regulates insulin sensitivity by activating the Tgfβ/Smads signaling pathway in AML12 cells. Analysis of AML12 cell lysates at the protein level revealed a significant increase in phosphorylated Smad2 expression when treated with sEV^AG^ containing high levels of Thbs1, indicating activation of the Thbs1/Tgfβ pathway “Fig. 5A”. Moreover, the protein interactions between Thbs1 and Tgfβ were enhanced in AML12 cells co-cultured with sEVAG compared to control and sEV^A^ co-cultured cells “Fig. 5B”. Furthermore, ITD-1, a pharmacological inhibitor of Tgfβ “Fig. 5C”, and LSKL as a Thbs1 antagonist “Fig. 5D”, reduced the levels of phosphorylated Smad2 in AML12 cells co-cultured with sEVAG. Additionally, we observed increased interactions between extracellular Thbs1 and the CD36 ligand, which plays a crucial role in lipid and glucose metabolism and promotes IR [27, 28]. Specifically, the interactions between Thbs1 and CD36 were elevated in AML12 cells co-cultured with sEV^AG^ compared to control and sEV^A^ “Fig. 5E”. However, the interaction between Thbs1 and CD36 was reduced by LSKL in AML12 cells co-cultured with sEV^AG^ “Fig. 5F”. Our findings propose an sEV^AG^-mediated mechanism of IR, potentially involving the activation of Tgfβ and CD36 pathways in recipient cells through secreted Thbs1.

### Inhibition of Thbs1 in ADSC/GDM-derived sEV improved insulin sensitivity

**Fig. 6:**
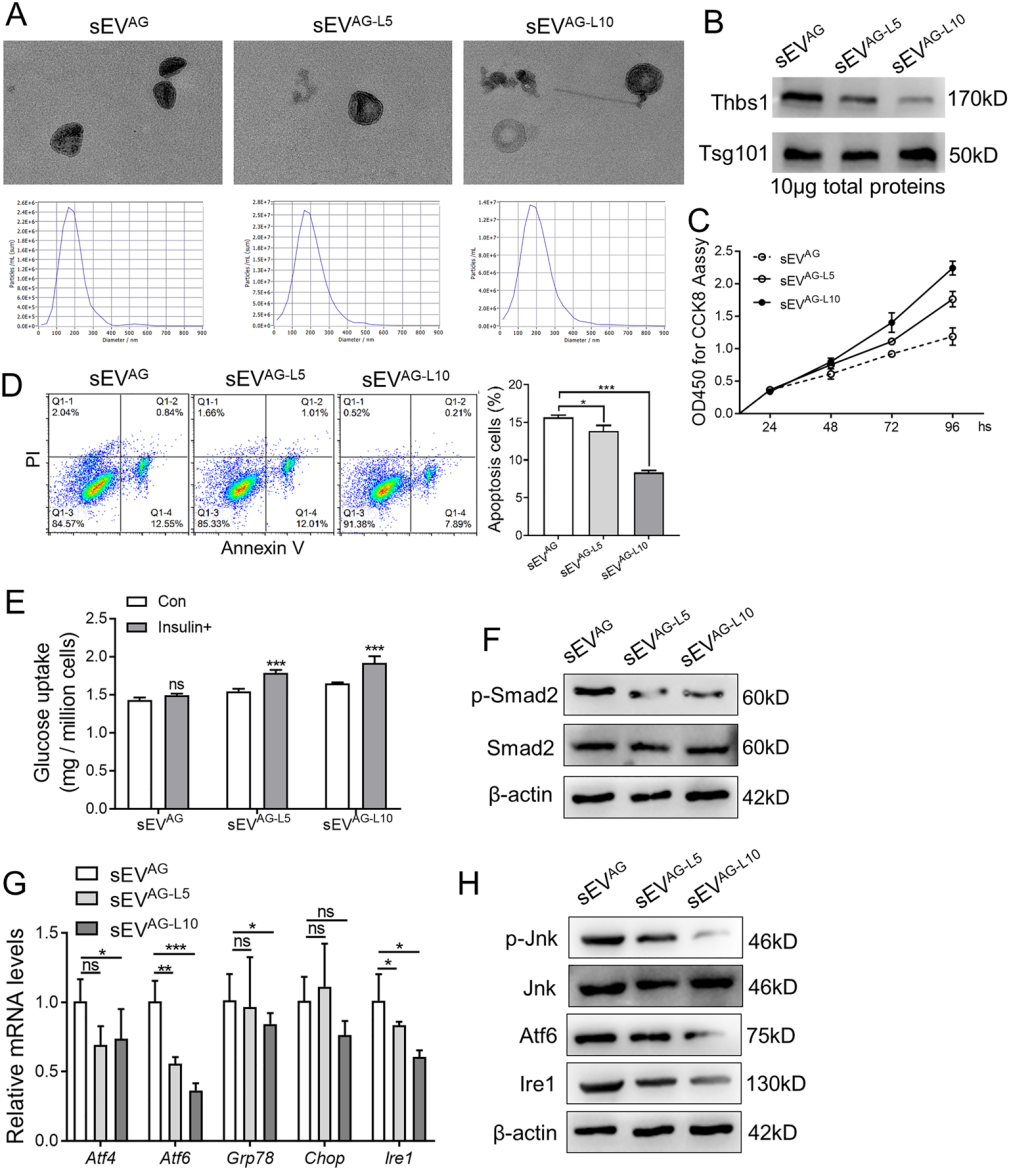
ADSC/GDM sEV restore insulin sensitivity to liver cells after Thbs1 pharmacological interference. **A** ADSC-GDM cells were treated with different concentrations of LSKL (0, 5 μM, 10 μM) for 72 h. sEV^AG^, sEV^AG−L5^ and sEV^AG−L10^ were collected and identified by TEM and NTA. **B** The expression of Thbs1 in sEV^AG^, sEV^AG−L5^ and sEV^AG−L10^ was identified by western blotting. **C** sEV^AG^, sEV^AG−L5^ and sEV^AG−L10^ were co-cultured with AML12 for 24–96 h, and cell viability was detected by CCK8 assays. **D** sEV^AG^, sEV^AG−L5^ and sEV^AG−L10^ were co-cultured with AML12 for 48 h, and cell apoptosis was detected by flow cytometry. **E** Glucose uptake of AML12 cells after insulin treatment was measured. F. sEV^AG^, sEV^AG−L5^ and sEV^AG−L10^ were co-cultured with AML12 for 48 h, and the expression of phosphorylated Smad2 and total Smad2 protein was detected by western blotting. **G**, **H** RT-PCR and western blotting were used to detect the expression of ER stress related genes in AML12 cells. Statistical data were presented as mean ± standard deviation (SD); ns, no significance; *P < 0.05, **P < 0.01, ***P < 0.001

To validate the role of Thbs1 in sEV^AG^, ADSC/GDM cells were treated with LSKL for 72 h, and sEV was isolated from the culture medium. The morphology and particle size distribution of sEV secreted by cells treated with 5 μM and 10 μM LSKL (sEV^AG−L5^ and sEV^AG−L10^) were examined using TEM and NTA “Fig. 6A”. Western blotting results demonstrated that the levels of Thbs1 protein in sEV^AG−L5^ and sEV^AG−L10^ were significantly lower than those in sEV^AG^ “Fig. 6B”. Co-culturing AML12 cells with sEV^AG−L5^ and sEV^AG−L10^ resulted in significantly increased cell viability “Fig. 6C”, decreased apoptosis “Fig. 6D”, and improved insulin sensitivity “Fig. 6E” compared to sEV^AG^. Mechanistically, co-culturing AML12 cells with sEV^AG−L5^ and sEV^AG−L10^ reduced the activation of the Tgfβ pathway, as evidenced by decreased expression of phosphorylated Smad2 “Fig. 6F”. Additionally, the mRNA and protein expression levels of ER stress-related genes were reduced in AML12 cells co-cultured with sEV^AG−L5^ and sEV^AG−L10^ “Fig. 6G and H”. Consequently, pharmacological inhibition of Thbs1 in sEV^AG^ aided in alleviating stress signals in mouse liver cells.

### Thbs1 pharmacological inhibition in vivo improves insulin sensitivity in GDM mice

**Fig. 7:**
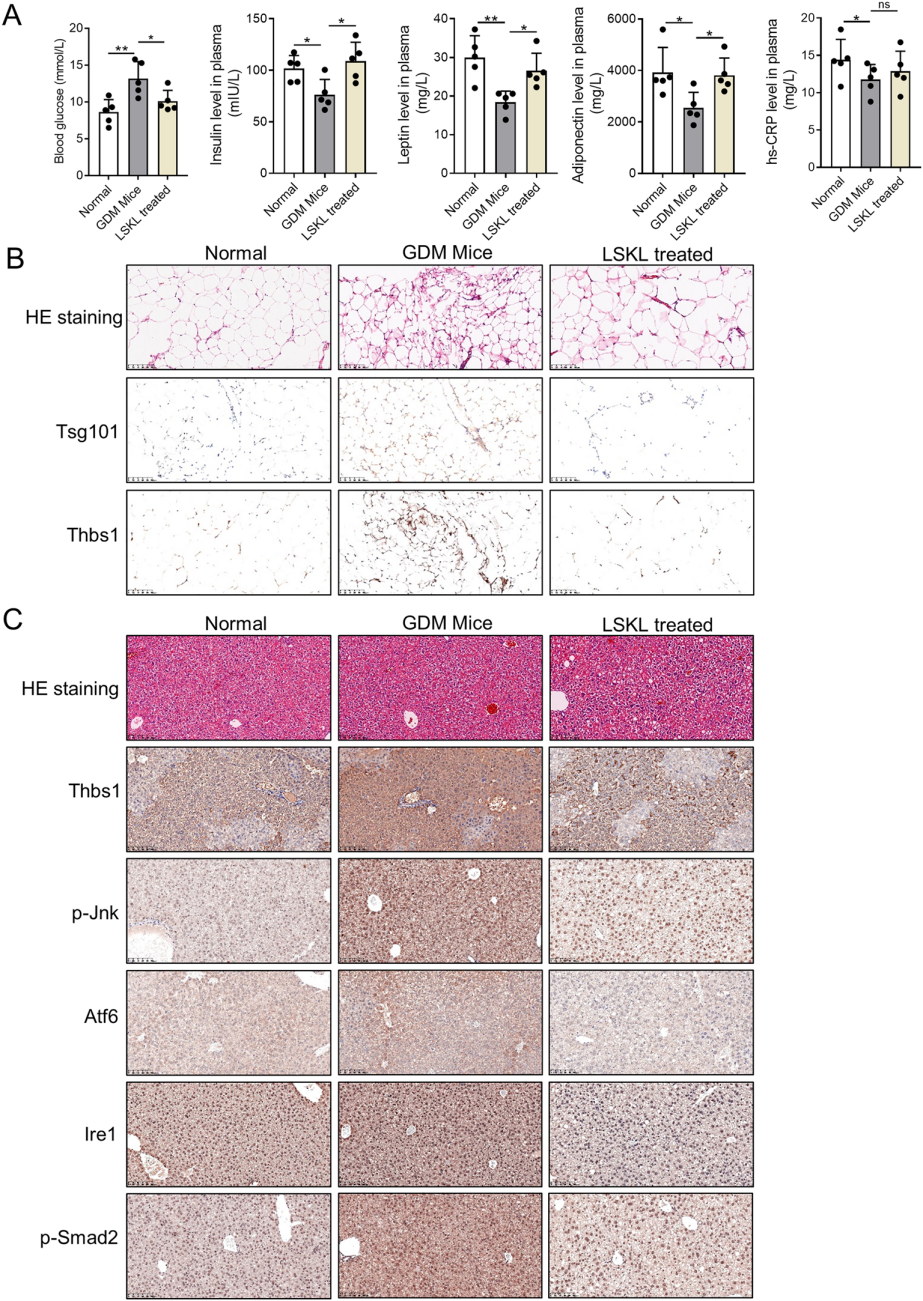
Targeted Thbs1 pharmacological therapy improves insulin resistance phenotype in GDM mice. **A** Plasma was extracted from normal, GDM, and LSKL-treated GDM mice, and blood glucose and insulin, leptin, adiponectin and hs-CRP levels were measured by glucose meter and ELISA kit. **B** HE staining and immunohistochemical detection of adipose tissues from normal, GDM, and LSKL treated mice. **C** HE staining and immunohistochemical detection of liver tissues from these mice. Statistical data were presented as mean ± standard deviation (SD); ns, no significance; *P < 0.05, **P < 0.01, ***P < 0.001

GDM mice were intraperitoneally injected with 1 mg/kg LSKL daily for 15 days before and after pregnancy. We evaluated the effects of LSKL treatment on insulin sensitivity and marker expression in tissues. The results indicated that LSKL treatment significantly reduced blood glucose levels and increased plasma insulin, leptin, and adiponectin levels in GDM mice “(Fig. 7A”. Immunological examination of abdominal and inguinal ATs, as well as liver tissues of GDM mice treated with either vehicle control or LSKL, revealed high expression of vesicular proteins Tsg101 and Thbs1 in the AT of GDM mice compared to normal mice. However, LSKL treatment attenuated the expression of Tsg101 and Thbs1 in the AT of GDM mice “Fig. 7B”. Immunohistochemical analysis demonstrated that the expression of Thbs1, ER stress regulatory proteins (phosphorylated Jnk, Atf6, Ire1), and phosphorylated Smad2 in the livers of GDM mice was higher than in normal mice. Nevertheless, LSKL treatment decreased their expression in the livers of GDM mice “Fig. 7C”. Hence, the peptide LSKL, targeting Thbs1, proved to be an effective pharmacological tool for improving IR in GDM mice.

## Discussion

In the present study, we aimed to investigate the underlying mechanisms of insulin sensitivity suppression induced by ADSCs in GDM mice, specifically focusing on sEVs. Our findings demonstrate that sEVs derived from ADSCs were responsible for the reduction of insulin sensitivity in normal tissues. Furthermore, we have provided evidence suggesting that sEVs derived from ADSCs of GDM mice carry Thbs1 protein, which inhibits cell viability and insulin sensitivity in liver cells. Mechanistically, the Thbs1 protein in sEVs interacts with CD36 and Tgfβ receptors in liver cells, activating the Tgfβ/Smad2 signaling pathway.

Obesity measurements before and during early pregnancy have been identified as strong predictors of fasting insulin concentration throughout pregnancy [29]. Pregnancy is associated with a disproportional accumulation of visceral fat and an increased risk of metabolic disease. Transient IR during pregnancy may contribute to intra-abdominal fat accumulation [30]. Increased fat expression and IR are significant features of pregnancy. Maternal obesity causes oxidative stress and metabolic dysfunction in both maternal and fetal health [30].

With defective regulation of adipocytokines in early-stage type 2 diabetes, GDM women represent an ideal research population, thereby enhancing the understanding of interrelationships [31]. Adiponectin, leptin, and high-sensitivity CRP have shown correlations with the onset of T2DM and microvascular complications [32]. Our findings revealed a significant decrease in insulin, leptin, adiponectin, and hs-CRP levels in the plasma of GDM mice at day 12 of gestation compared to normal gestational mice. These results aligned with various cross-sectional reports [33, 34]. Furthermore, placenta-produced leptin has been associated with weight regulation and metabolism, with reported levels being both elevated [35] and within normal ranges [36]. Notably, it seems that HsCRP does not significantly contribute to pregnancy-induced insulin resistance in GDM or in women with a healthy pregnancy [31]. A study involving 180 women found reduced adiponectin levels in GDM [37]. Additionally, previous reports indicate that when BMI and adiposity were considered, hsCRP was not significantly associated with GDM [38]. Therefore, the observed elevation of hsCRP is not deemed an important cause or consequence of reduced IR during pregnancy [31]. Our research outcomes validated the findings of the above studies.

GDM is characterized by increased adipose expression and secretion of proinflammatory cytokines. However, the mechanism behind these changes remains unclear. Previous studies have shown that GDM women release significantly higher levels of AT-derived exosomes compared to healthy pregnant women. These exosomes increase the expression of glucose metabolism-related factors in placental cells, thereby mediating IR formation [39]. ADSCs, somatic stem cells obtained from white AT [40], produce adipocytokines that play endocrine and paracrine roles and participate in the occurrence of IR [41]. The release of sEVs, observed in many pathological conditions [42, 43], including T2DM and GDM [44, 45], is higher in patients with GDM compared to patients with normal glucose tolerance. AT from women with GDM releases a greater number of sEVs compared to women with normal glucose tolerance, supporting our research findings that hyperglycemia can induce the secretion of sEVs from AT. However, the relationship between sEVs secreted by ADSCs and GDM-induced IR remains unclear.

ADSCs have gained significant attention in regenerative medicine and stem cell therapy due to their ability to repair tissues and regulate immunity. However, studies have shown that under continuous high glucose environments, the glucose metabolism, cell replication, apoptosis, and differentiation ability of ADSCs are impaired, with a more pronounced negative impact on ADSCs derived from patients with diabetes [46]. Liver tissue can be damaged in the hyperglycemic environment of diabetes, leading to liver fibrosis. Addition of hepatocyte growth factors to ADSCs has been proposed as a method to treat liver fibrosis in diabetic patients [47]. Our study showed differences in the biological functions of AML-12 cells between sEV^A^ and sEV^AG^. Compared to sEV^A^, sEV^AG^ significantly inhibited cell proliferation, promoted cell apoptosis, and enhanced IR under insulin induction, consistent with the findings from existing literature.

ER related- kinase, inositol demand enzyme 1α (IRE1-α) existing in mammalian endoplasmic, and reticulum membrane activating transcription factor 6 (ATF-6), are three ER transmembrane protein, playing important roles in the signal transduction pathway of ERS. In addition, X-box binding protein-1(XBP1) and activating transcription factor 4 (Atf4) are also crucial in inducing ERS response and can regulate genes involved in ERS [48]. ERS plays a core role in triggering IR and T2DM [19]. ERS in liver histiocytes can cause changes in insulin signaling pathways, leading to IR through overactivation of the c-Jun amino terminal kinase pathway and serine phosphorylation of insulin receptor substrate 1 [49]. There is also a connection between extracellular vesicles, ERS, and IR. For example, extracellular vesicles derived from bone marrow stem cells can significantly inhibit insulin receptor substrate induced by IR in a renal ischemia–reperfusion model [50]. Additionally, under pathological conditions, abnormally expressed extracellular vesicles can induce cellular ERS response, leading to cell apoptosis and tissue dysfunction [51]. Therefore, it is speculated that extracellular vesicles derived from ADSCs induce the expression of ERS signaling-related proteins in liver tissue cells, thereby impairing insulin sensitivity and promoting the formation of IR in GDM. Our study demonstrated that sEV^AG^ increased the expression of ERS signaling-related proteins Atf4 and Atf6 in AML-12 cells, thereby damaging cellular insulin sensitivity. Several studies have suggested that ERS is a core mechanism underlying various diseases, including IR and T2DM [19]. The overactivation of multiple ERS signal-related genes, such as ATF4 and ATF6, in AT [52]. Therefore, the activation of ERS signals plays a crucial role in the occurrence of IR and hyperglycemia. This study shows that sEV^AG^ increased the expression of ERS signaling related proteins ATFf4 and ATF6 in AML-12 cells, damaging the insulin sensitivity of cells. However, further research is needed to understand how ERS signaling-related proteins Atf4 and Atf6 promote IR in AML-12 cells.

Thbs1 is abundantly found in alpha granules of platelets, but normal plasma levels are typically low (100–200 ng/ml). The expression of Thbs1 increases in high glucose and high-fat environments. It is also elevated in T2DM and cardiovascular disease [53]. In the disease model of visceral fat and IR, Thbs1 is highly expressed in the visceral AT of obese rats [54]. Our results revealed specific expression of Thbs1 in the exosomes derived from ADSCs of GDM mice. Knockout of the Thbs1 gene can protect mouse AT from inflammation and invasion of IR [18]. This study demonstrated that high expression of Thbs1 in sEV^AG^ damages cellular insulin sensitivity, while the addition of Thbs1 peptide antagonist LSKL can restore insulin sensitivity, consistent with existing research findings.

## Conclusion

Overall, our results suggest that sEVs are responsible for inducing IR in normal tissues mediated by ADSCs in GDM mice. Treatment with exo-GDM inhibited cell viability and insulin sensitivity in normal mouse liver cells compared to exo-NGT. Additionally, exo-GDM amplified the expression of Thbs1 proteins through the activation of Tgfβ/Smad2 signaling in ADSC/GDM-derived sEVs. These findings align with previous studies demonstrating the ability of sEVs to interact with and regulate gene and miRNA expression [55, 56], stimulate cytokines, and impair insulin response in target cells [57]. To the best of our knowledge, this is the first study to propose ADSC/GDM-derived sEVs as "switches" that upregulate Thbs1 and induce the increase of ERS signaling-related proteins Atf4 and Atf6 in AML-12 cells, thereby damaging cellular insulin sensitivity and promoting the formation of IR in GDM mice. This study provides a foundation for further understanding the molecular mechanisms underlying the formation of insulin resistance in GDM and may offer new strategies for targeted treatment of GDM and even T2DM.

### Supplementary Information

**Additional file 1: Figure S1.** Immunofluorescence analysis revealed the positive expression of Cd44 and Cd29, the mesenchymal stem cell markers, in isolated primary ADSCs and ADSC/GDMs.

**Additional file 2: Figure S2.** The monocyte population marker Cd14 showed negative expression in both ADSCs and ADSC/GDMs.

**Additional file 3. Supplementary Table 1.** Primer sequences for RT-PCR.

**Additional file 4. Supplementary Table 2.** Differentially expressed proteins in sEVAG and sEVA group.

## Data Availability

The original contributions presented in the study are included in the article/supplementary material. Further inquiries can be directed to the corresponding author.
