# Supplementary figures and images for: Adipose stem cells-derived small extracellular vesicles transport Thrombospondin 1 cargo to promote insulin resistance in gestational diabetes mellitus
Source: Diabetol Metab Syndr. 2024 May 19;16:105. doi: 10.1186/s13098-024-01276-1 (PMC11103858; doi:10.1186/s13098-024-01276-1)

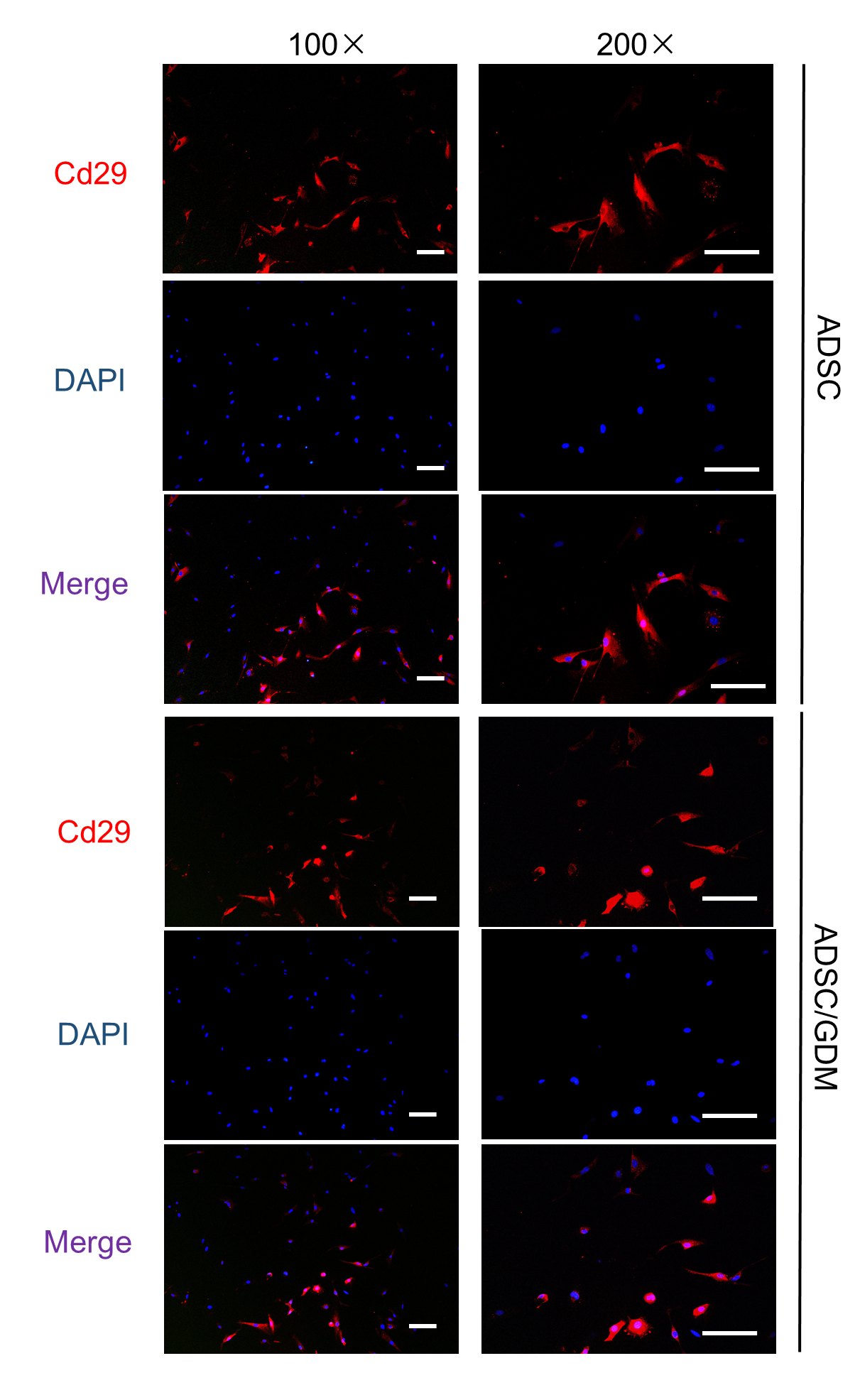

Supplement: Supplementary file 1 — Additional file 1: Figure S1. Immunofluorescence analysis revealed the positive expression of Cd44 and Cd29, the mesenchymal stem cell markers, in isolated primary ADSCs and ADSC/GDMs. [file 13098_2024_1276_MOESM1_ESM.tif]

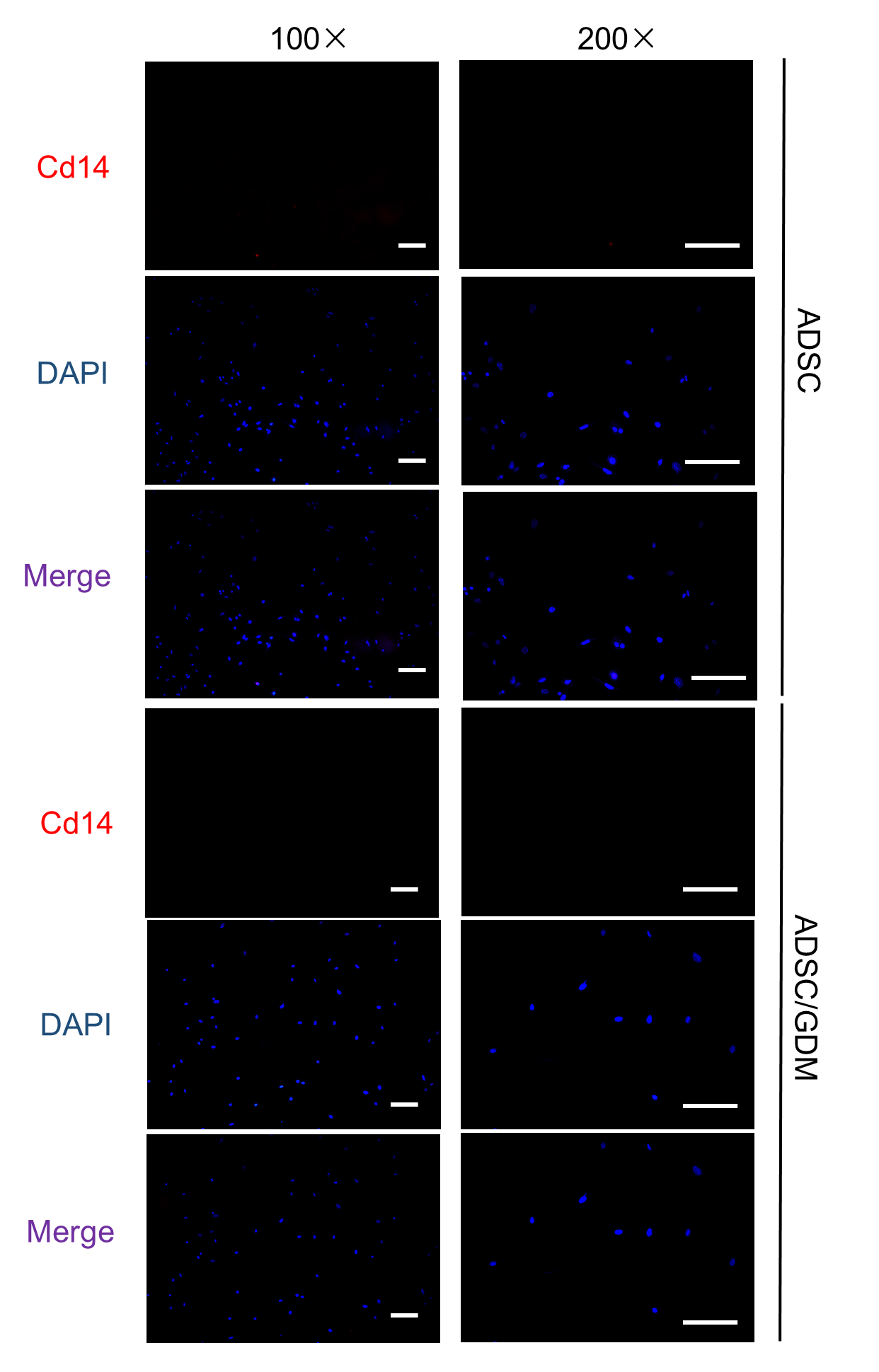

Supplement: Supplementary file 2 — Additional file 2: Figure S2. The monocyte population marker Cd14 showed negative expression in both ADSCs and ADSC/GDMs. [file 13098_2024_1276_MOESM2_ESM.tif]
